# Transforming Growth Factorβ1 Overexpression Is Associated with Insulin Resistance and Rapidly Progressive Kidney Fibrosis under Diabetic Conditions

**DOI:** 10.3390/ijms232214265

**Published:** 2022-11-17

**Authors:** Valeria Fridman D’Alessandro, Atsuro Takeshita, Taro Yasuma, Masaaki Toda, Corina N. D’Alessandro-Gabazza, Yuko Okano, Suphachai Tharavecharak, Chisa Inoue, Kota Nishihama, Hajime Fujimoto, Tetsu Kobayashi, Yutaka Yano, Esteban C. Gabazza

**Affiliations:** 1 Department of Immunology, Mie University Faculty and Graduate School of Medicine, Edobashi 2-174, Tsu 514-8507, Japan; 2 Department of Diabetes and Endocrinology, Mie University Faculty and Graduate School of Medicine, Edobashi 2-174, Tsu 514-8507, Japan; 3 Department of Pulmonary and Critical care Medicine, Mie University Faculty and Graduate School of Medicine, Edobashi 2-174, Tsu 514-8507, Japan

**Keywords:** insulin resistance, diabetes mellitus, transforming growth factorβ, glucose intolerance

## Abstract

Diabetes mellitus is a global health problem. Diabetic nephropathy is a common complication of diabetes mellitus and the leading cause of end-stage renal disease. The clinical course, response to therapy, and prognosis of nephropathy are worse in diabetic than in non-diabetic patients. The role of transforming growth factorβ1 in kidney fibrosis is undebatable. This study assessed whether the overexpression of transforming growth factorβ1 is associated with insulin resistance and the rapid progression of transforming growth factorβ1-mediated nephropathy under diabetic conditions. Diabetes mellitus was induced with streptozotocin in wild-type mice and transgenic mice with the kidney-specific overexpression of human transforming growth factorβ1. Mice treated with saline were the controls. Glucose tolerance and kidney fibrosis were evaluated. The blood glucose levels, the values of the homeostasis model assessment for insulin resistance, and the area of kidney fibrosis were significantly increased, and the renal function was significantly impaired in the diabetic transforming growth factorβ1 transgenic mice compared to the non-diabetic transgenic mice, diabetic wild-type mice, and non-diabetic mice. Transforming growth factorβ1 impaired the regulatory effect of insulin on glucose in the hepatocyte and skeletal muscle cell lines. This study shows that transforming growth factorβ1 overexpression is associated with insulin resistance and rapidly progressive kidney fibrosis under diabetic conditions in mice.

## 1. Introduction

The increased number of diabetes mellitus (DM) patients is a serious health problem and a great economic burden worldwide [1]. According to recent epidemiological data, the global population of DM patients is almost 500 million [2]. DM is associated with high morbidity and mortality rates. The deaths globally attributable to DM were about 1.5 million in 2019 [3]. Microangiopathy (retinopathy, nephropathy, neuropathy) and macroangiopathy (stroke, coronary ischemic diseases, lower extremity arterial disease) are the main causes of high morbidity and death rates [4,5,6]. Of these, diabetic nephropathy is one of the most common complications and the most frequent cause of chronic kidney disease and end-stage renal disease [7]. The clinical course, response to therapy, and prognosis of nephropathy are worse in patients with DM than in non-diabetic patients [8,9,10]. The incidence of diabetic nephropathy is 20 to 40% in the American diabetic population [7,11]. In Japan, 43.2% of all-cause end-stage renal disease is associated with diabetic nephropathy [12]. The functional and histopathological characteristics of diabetic nephropathy include proteinuria, enhanced cell proliferation and matrix deposition in the mesangium, glomerulosclerosis, and interstitial fibrosis with a decreased glomerular filtration rate [13].

Transforming growth factor (TGF)β1 is a multifunctional cytokine that can promote fibrogenesis by increasing the production of extracellular matrix components (e.g., collagen, fibronectin, periostin, elastin) by fibroblasts or myofibroblasts, stimulating the platelet-derived growth-factor-mediated proliferation and recruitment of fibroblasts, accelerating the epithelial–mesenchymal transition, or suppressing the expression of collagen-degrading matrix metalloproteinases [11,14,15,16,17]. Several cases of evidence have proved the critical role that TGFβ1 plays in the pathogenesis of diabetic nephropathy [11,18,19]. The circulating levels of TGFβ1 are significantly increased in diabetic nephropathy and are significantly correlated with the severity of renal dysfunction [11,20,21]. Patients with diabetic nephropathy also show high urinary levels of TGFβ1, and mice with the glomerulus-specific overexpression of human TGFβ1 develop chronic kidney disease and renal failure [11,18,21]. TGFβ1 exerts its effects by binding to the cell surface receptors and activating the SMAD transcription factors [14]. In addition to stimulating the fibrosis process, recent studies suggest that the TGFβ1/SMAD signal pathway is also implicated in the pathogenesis of metabolic disorders, including obesity and DM [22]. Patients with morbid obesity and diabetic nephropathy showed a high expression level of TGFβ1, and an enhanced level of TGFβ1 was reported as a risk factor for type-2 DM [23,24,25]. Evidence also shows that the inhibition of the TGFβ1/SMAD pathway protects the body against diet-induced obesity and DM and that the TGFβ1 level is significantly correlated with adipose deposition in experimental animals and human subjects [26].

In the present study, we hypothesized that the overexpression of TGFβ1 is associated with insulin resistance and the rapid progression of TGFβ1-mediated nephropathy under diabetic conditions. To demonstrate this hypothesis, we compared the development of insulin resistance, DM, and the progression of diabetic nephropathy between diabetic and non-diabetic transgenic (TG) mice overexpressing human TGFβ1 in the kidneys and wild-type (WT) mice. 

## 2. Results

### 2.1. Increased Circulating Levels of Active TGFβ1 in Diabetic TGFβ1 TG Mice

**Figure 1:**
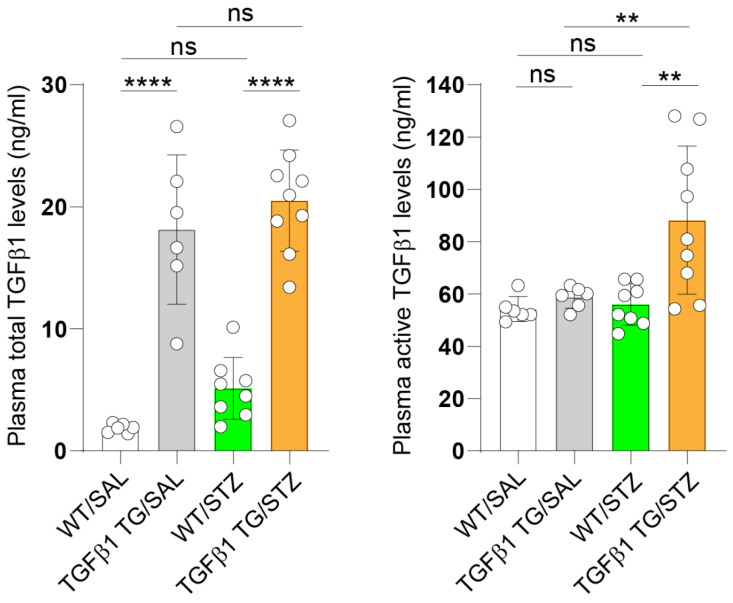
Increased circulating levels of total and active TGFβ1 in TGFβ1 TG mice with diabetes mellitus. The plasma levels of total and active TGFβ1 were measured by immunoassay using a commercial kit. The number of mice were as follows: WT/SAL *n* = 6, TGFβ1 TG/SAL *n* = 6, WT/STZ *n* = 8, TGFβ1 TG/STZ *n* = 9. Data are expressed as mean ± S.D. Statistical analysis by one-way analysis of variance (ANOVA) with Newman–Keuls’ test. ** *p* < 0.01; **** *p* < 0.0001; ns, not significant.

As expected, the plasma levels of total TGFβ1 were significantly increased in the diabetic (TGFβ1 TG/STZ) and non-diabetic (TGFβ1 TG/SAL) TGFβ1 TG mice compared to their non-diabetic (WT/SAL) and diabetic (WT/STZ) WT counterparts. On the other hand, the plasma levels of the active form of TGFβ1 were significantly increased in the diabetic TGFβ1 TG mice compared to the non-diabetic TGFβ1 TG mice and non-diabetic and diabetic WT mice (Figure 1). 

### 2.2. Increased Blood Glucose Levels and Abnormal Intraperitoneal Glucose Tolerance Test in Diabetic TGFβ1 TG Mice

**Figure 2:**
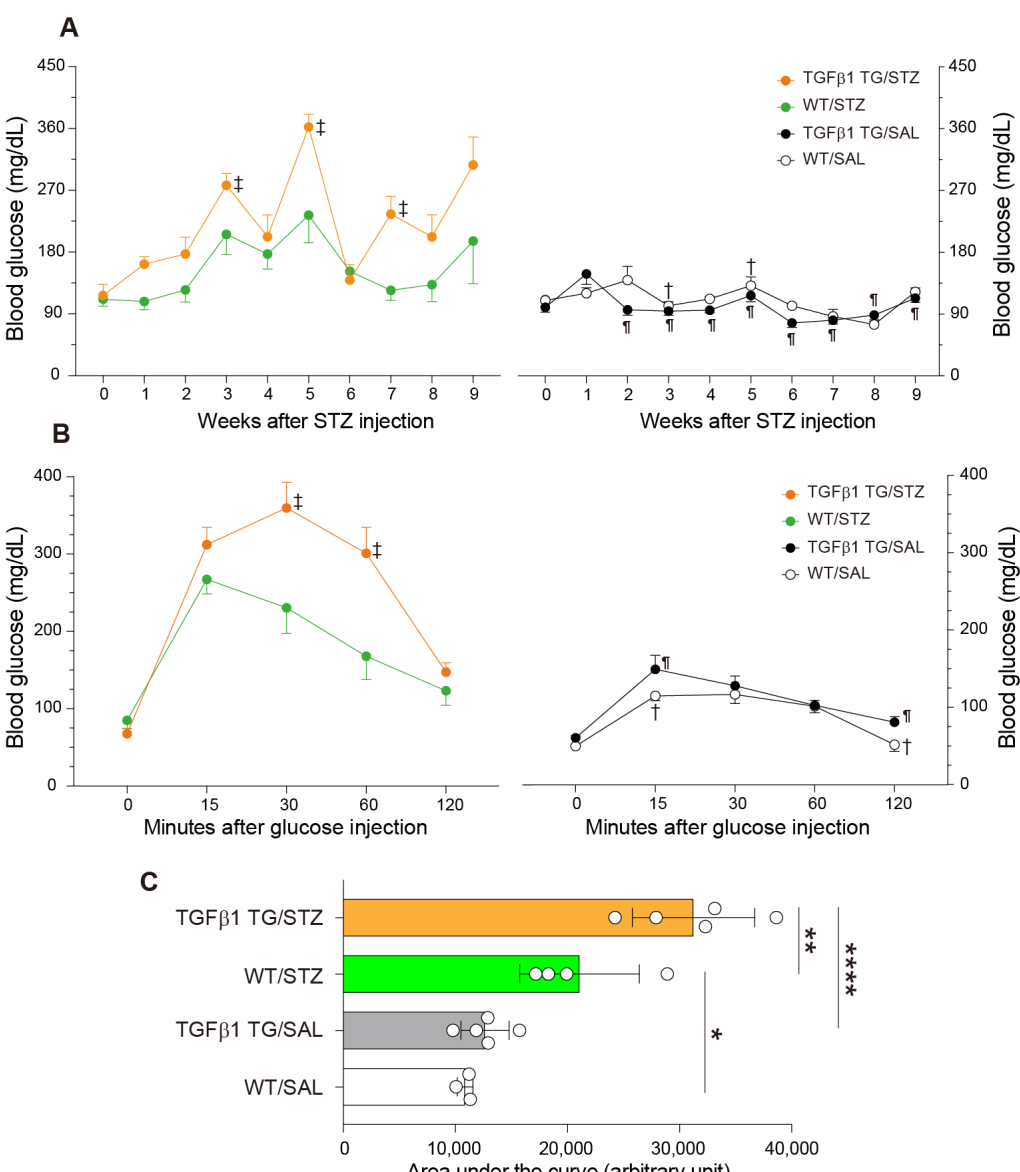
Mice overexpressing transforming growth factorβ1 show insulin resistance. Mice received intraperitoneal injections of streptozotocin (STZ) for five days and were then sacrificed after 9 weeks. The control mice received intraperitoneal injections of saline for the same interval and schedule. (**A**) Blood glucose levels were measured every week for 9 weeks, as described in the Materials and Methods. (**B**) An intraperitoneal glucose tolerance test was performed, as described in the Materials and Methods. (**C**) The area under the curve during the intraperitoneal glucose tolerance test was calculated. Number of mice: *n* = 3 in WT/SAL, *n* = 5 in TGFβ1 TG/SAL, *n* = 4 in WT/STZ, *n* = 5 in TGFβ1 TG/STZ. Data are the mean ± S.D. Statistical analysis by ANOVA with Newman–Keuls’ test or Kruskal–Wallis ANOVA with Dunn’s test. ¶ *p* < 0.05 vs. TGFβ1 TG/STZ; ‡ *p* < 0.05 vs. WT/STZ; † *p* < 0.05 vs. WT/STZ; * *p* < 0.05; ** *p* < 0.01; **** *p* < 0.0001.

The glucose levels in the blood were longitudinally measured every week for nine weeks after STZ intraperitoneal injection. The blood concentration of glucose was significantly enhanced in the TGFβ1 TG mice with DM at weeks 3, 5, and 7 after the STZ injection compared to their counterpart WT mice with DM and compared to the non-diabetic TGFβ1 TG mice at weeks 2, 3, 4, 5, 6, 7, 8, and 9. In addition, the blood glucose levels were significantly increased in the diabetic WT mice at weeks 3 and 5 compared to the non-diabetic WT mice (Figure 2A).

The intraperitoneal glucose tolerance test revealed a significant increase in the blood glucose levels of the diabetic TGFβ1 TG mice after 30 and 60 min of glucose injection compared to their counterpart diabetic WT mice and after 15 and 120 min of glucose injection compared to the non-diabetic TGFβ1 TG mice. In addition, the blood glucose levels were significantly increased in the diabetic WT mice after 15 and 120 min of glucose injection compared to the non-diabetic WT mice (Figure 2B). The blood glucose levels were similar at the starting point of the intraperitoneal glucose tolerance test. Similar levels of blood glucose at the starting point of the intraperitoneal glucose tolerance test in the STZ model have also been reported [27,28,29,30,31,32]. Our calculation of the area under the curve also disclosed a significant difference in the blood glucose levels between the mice treated with streptozotocin (STZ), the TGFβ1 TG/STZ and WT/STZ mice, and the TGFβ1 TG/STZ and TGFβ1 TG/SAL (saline) mice, and between the WT/STZ and WT/SAL mice (Figure 2C).

### 2.3. Reduced Plasma Insulin Levels after Glucose Injection and Insulin Resistance in Diabetic TGFβ1 TG Mice

**Figure 3:**
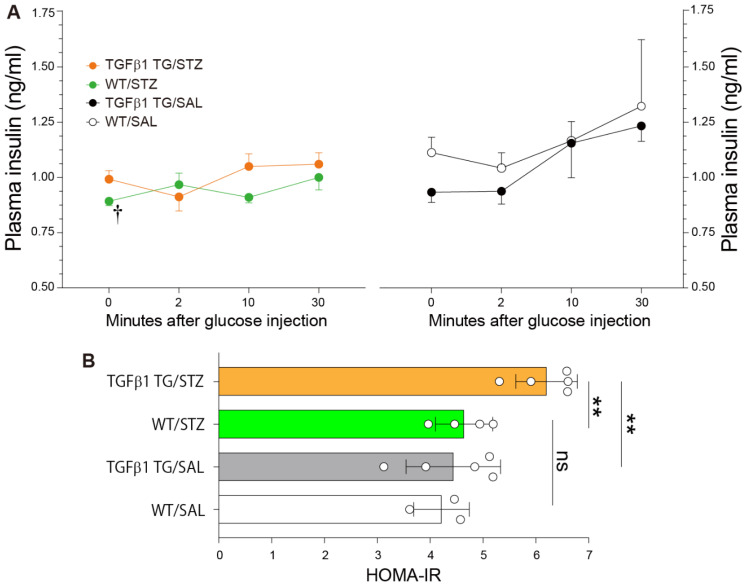
Reduced plasma insulin levels after glucose injection and insulin resistance in diabetic TGFβ1 TG mice. (**A**) The glucose-stimulated insulin secretion test was performed after 16 h of fasting by the intraperitoneal injection of glucose. Insulin was measured using blood samples taken after 0, 2, 10, and 30 min of glucose injection. (**B**) Homeostasis model assessment for insulin resistance (HOMA-IR) was determined before glucose administration, as described in the Materials and Methods. Number of mice: *n* = 3 in WT/SAL, *n* = 5 in TGFβ1 TG/SAL, *n* = 4 in WT/STZ, *n* = 5 in TGFβ1 TG/STZ. Data are the mean ± S.D. Statistical analysis by Kruskal–Wallis ANOVA with Dunn’s test. † *p* < 0.05 vs. WT/SAL; ** *p* < 0.01; ns, not significant.

The glucose-stimulated insulin secretion test was performed after fasting. The plasma insulin level was significantly reduced in the WT/STZ mice compared to the WT/SAL mice after 10 min of glucose injection. The plasma insulin level was also reduced in the WT/STZ group compared to the WT/SAL group after 30 min of glucose injection, although the reduction was not statistically significant (Figure 3A). The plasma insulin level was also significantly decreased in the TGFβ1 TG/STZ group compared to the TGFβ1 TG/SAL group after 10 and 30 min of glucose injection, although the decrease in the insulin levels was not statistically significant. However, the calculated values of the homeostasis model assessment for insulin resistance (HOMA-IR) were significantly increased in the diabetic TGFβ1 TG mice compared to the diabetic WT mice and non-diabetic TGFβ1 TG mice. No significant difference was observed between the WT/STZ and WT/SAL groups (Figure 3B).

### 2.4. Reduced Area of Pancreatic Islets in Diabetic TGFβ1 TG Mice

**Figure 4:**
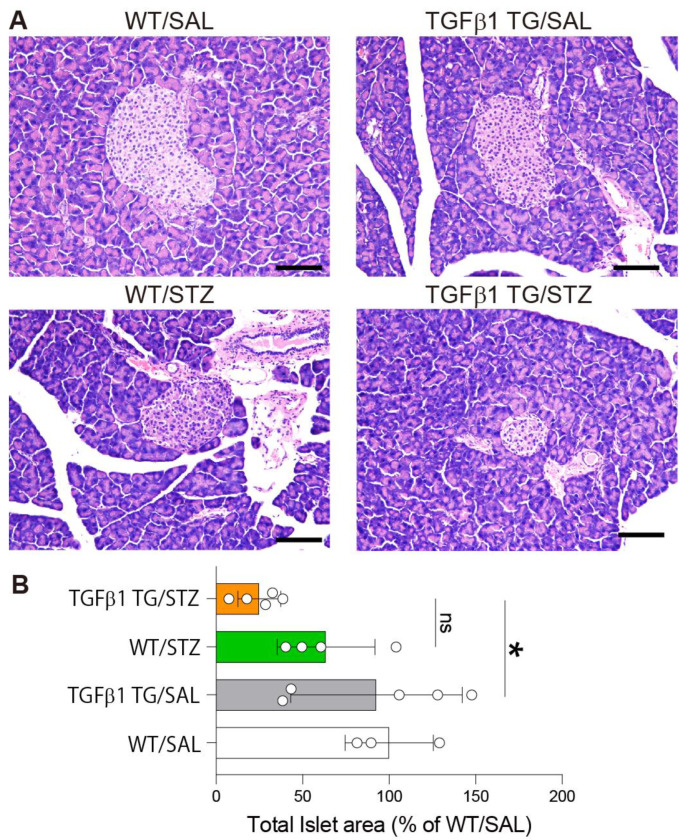
Diabetic transforming growth factorβ1 mice have a decreased area of pancreatic islets. (**A**) Mice were sacrificed in week 9 after streptozotocin or saline intraperitoneal injection. The pancreas was incised, removed, and prepared for hematoxylin and eosin staining. (**B**) Area of pancreatic islets measured in each group using the WinROOF image processing software. Number of mice: WT/SAL *n* = 3, TGFβ1 TG/SAL *n* = 5, WT/STZ *n* = 4, TGFβ1 TG/STZ *n* = 5. Scale bars indicate 200 µm. Data are the mean ± S.D. Statistical analysis by ANOVA with Newman–Keuls’ test. * *p* < 0.05; ns, not significant.

The pancreas was resected and stained with hematoxylin and eosin to measure the islet areas using the WinROOF image software. The area of the islets was significantly reduced in the TGFβ1 TG/STZ mice compared to the TGFβ1 TG/SAL mice. However, no significant difference was observed between the TGFβ1 TG/STZ and WT/STZ mice (Figure 4A,B).

### 2.5. TGFβ1 Impaired Insulin Action in the Hepatocyte and Skeletal Muscle Cell Lines

**Figure 5:**
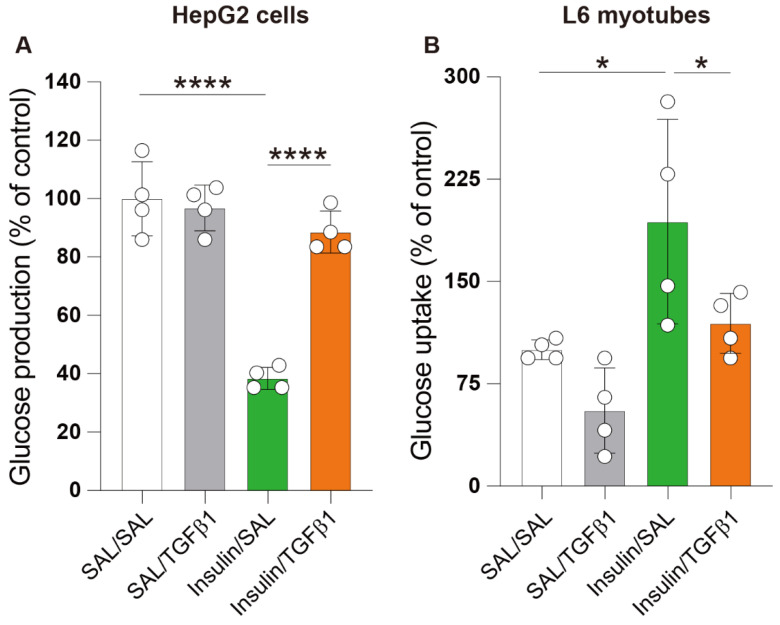
Transforming growth factorβ1 worsens insulin sensitivity in target cells. HepG2 (**A**) and L6 myoblasts (**B**) were cultured in the presence of insulin alone (200 nM) or in the presence of both insulin and transforming growth factorβ1 alone (20 ng/mL) for 4 h before measuring the medium glucose levels. Data are expressed as mean ± S.D. *n* = 4 per group. Statistical analysis by ANOVA with Newman–Keuls’ test. * *p* < 0.05; **** *p* < 0.0001.

Under insulin resistance conditions, the insulin-mediated suppression of glucose production by the hepatocytes and uptake of glucose by the skeletal muscles is impaired. In the present study, we used HepG2 cells and L6 myotubes to evaluate whether TGFβ1 induces insulin resistance in vitro [33]. HepG2 cells and L6 myotubes were cultured in the presence of insulin, and the effect of TGFβ1 on the glucose levels in the cell supernatants was evaluated. The presence of TGF-β1 markedly reversed the reduction in glucose release by the HepG2 cells with insulin (Figure 5A). On the other hand, the significantly increased glucose uptake induced by insulin in the L6 cells was significantly inhibited by TGFβ1 (Figure 5B).

### 2.6. Progressive Renal Dysfunction in TGFβ1 TG Mice with DM

**Figure 6:**
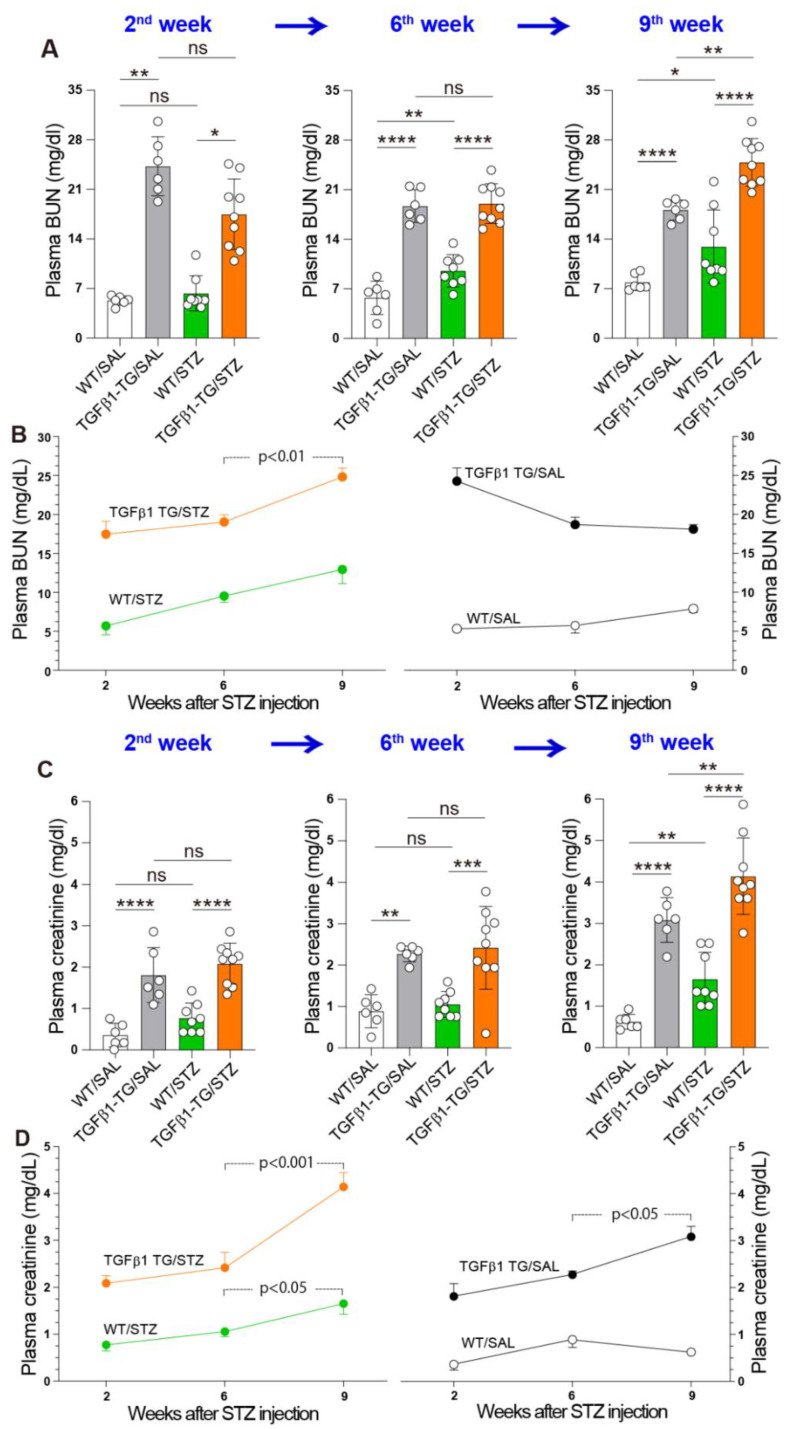
Progressive renal dysfunction in diabetic transforming growth factorβ1 transgenic mice. (**A**) Blood urea nitrogen (BUN) was measured by colorimetric methods, and creatinine was measured by an enzymatic method. (**B**) Weekly changes in plasma BUN. (**C**) Plasma creatinine was measured by an enzymatic method. (**D**) Weekly changes in plasma creatinine. The number of mice for the plasma evaluation: WT/SAL *n* = 6, TGFβ1 TG/SAL *n* = 6, WT/STZ *n* = 8, TGFβ1 TG/STZ *n* = 9. Data are expressed as the mean ± S.D. Statistical analysis by ANOVA with Newman–Keuls’ test or Kruskal–Wallis ANOVA with Dunn’s test. * *p* < 0.05; ** *p* < 0.01; *** *p* < 0.001; **** *p* < 0.0001. WT, wild-type mice, TGFβ1 TG, transforming growth factor-β1 transgenic mice.

The blood urea nitrogen (BUN) and creatinine plasma levels were measured at weeks 2, 6, and 9 after the induction of DM with STZ. The non-diabetic TGFβ1 TG (TGFβ1 TG/SAL) mice showed significantly increased plasma levels of BUN compared to their non-diabetic WT (WT/SAL) counterparts at weeks 2, 6, and 9. The plasma levels of BUN were significantly increased in the diabetic TGFβ1 TG (TGFβ1 TG/STZ) mice compared to the diabetic WT (WT/STZ) mice at weeks 2, 6, and 9 after the induction of DM with STZ. The diabetic TGFβ1 TG mice showed no significant change in the plasma levels of BUN at weeks 2 and 6 after STZ injection, but the levels were significantly increased in the diabetic TGFβ1 TG mice compared to the non-diabetic TGFβ1 TG mice at week 9 after STZ injection. The plasma BUN levels were significantly increased at weeks 6 and 9 after STZ injection in the diabetic WT compared to the non-diabetic WT group (Figure 6A).

The comparative evaluation of the plasma levels of BUN over the weeks showed a significant increase in the plasma levels of BUN at week 9 compared to week 6 in the diabetic TGFβ1 TG mice. The plasma BUN levels were also increased at week 9 compared to week 6 in the diabetic WT mice, but the increase was not statistically significant. There were no significant changes in the plasma BUN levels between weeks 6 and 9 in the non-diabetic TGFβ1 TG and non-diabetic WT mice (Figure 6B).

The plasma concentrations of creatinine were significantly increased in the diabetic TGFβ1 TG mice at weeks 2, 6, and 9 after STZ injection compared to their diabetic WT counterparts. The plasma creatinine levels were not significantly different between the diabetic and non-diabetic TGFβ1 TG mice at weeks 2 and 6 after STZ injection but significantly increased in the diabetic TGFβ1 TG mice compared to the non-diabetic TGFβ1 TG mice at week 9 after the induction of DM with STZ. The non-diabetic TGFβ1 TG mice showed significantly elevated plasma levels of creatinine at weeks 2, 6, and 9 after STZ injection compared to the non-diabetic WT counterparts. The plasma creatinine levels were significantly increased in the diabetic WT mice compared to the non-diabetic WT mice 9 weeks after the induction of DM with STZ (Figure 6C).

The comparative evaluation of the plasma levels of creatinine over the weeks showed a significant and strong increase in the plasma levels of creatinine oat week 9 compared to week 6 in the diabetic TGFβ1 TG mice. The plasma creatinine levels in the diabetic WT mice were also increased at week 9 compared to week 6, but the increase was weak. The plasma creatinine levels in the non-diabetic TGFβ1 TG mice were also weakly increased at week 9 compared to week 6 (Figure 6D).

### 2.7. Progressive Glomerulosclerosis in Diabetic TGFβ1 TG Mice

**Figure 7:**
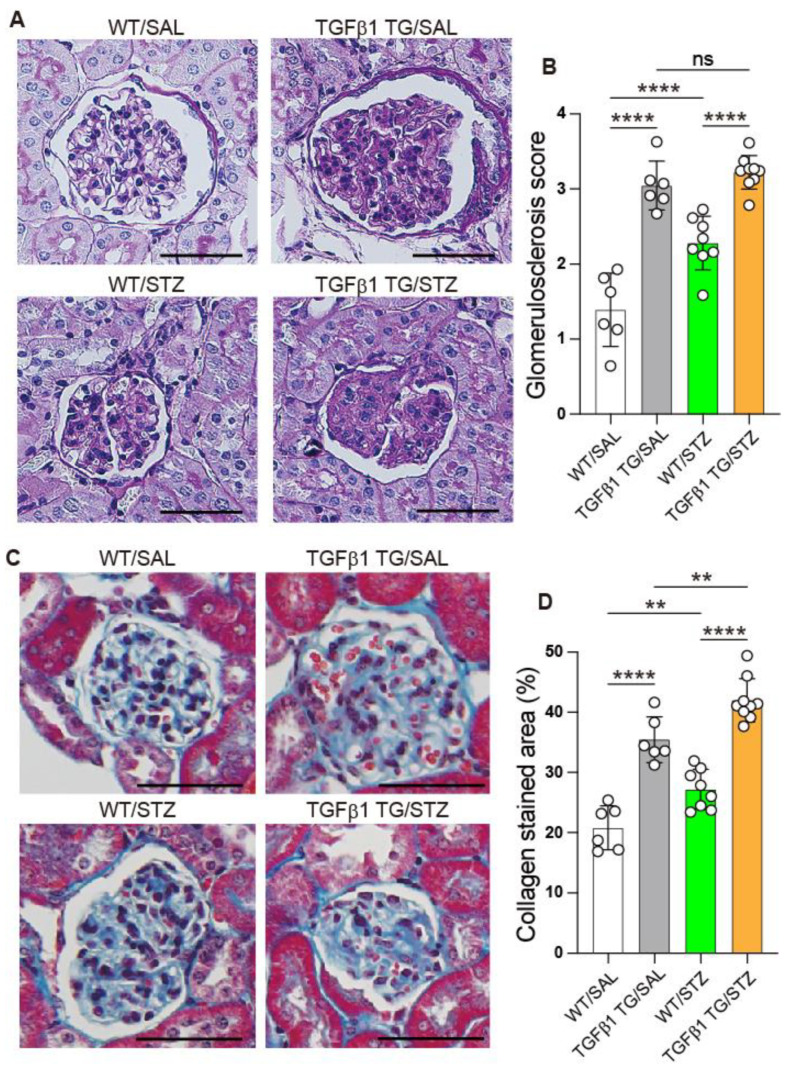
Progressive glomerulosclerosis in transforming growth factorβ1 transgenic mice with diabetes mellitus. Renal tissues were cut and stained with periodic acid–Schiff (**A**,**B**) or Masson’s trichrome (**C**,**D**) and then quantified using a scoring system or the WinROOF imaging software. Scale bars indicate 50 µm. Number of mice: WT/SAL *n* = 6, TGFβ1 TG/SAL *n* = 6, WT/STZ *n* = 8, TGFβ1 TG/STZ *n* = 9. Statistical analysis by ANOVA with Newman–Keuls’ test. ** *p* < 0.01; **** *p* < 0.0001. WT, wild-type mice, TGFβ1 TG, transforming growth factorβ1 transgenic mice.

Renal tissue specimens were cut and stained with periodic acid–Schiff and Masson’s trichrome to evaluate the fibrosis grade of each group. The tissue slides were used for the scoring of glomerulosclerosis. The glomerulosclerosis score was not significantly increased in the diabetic TGFβ1 TG mice compared to the non-diabetic TGFβ1 TG mice but significantly increased in the diabetic TGFβ1 TG mice compared to the diabetic WT mice. The non-diabetic TGFβ1 TG mice and diabetic WT mice showed significantly higher glomerulosclerosis scores than the non-diabetic WT mice (Figure 7A,B). The glomerular area stained with collagen (trichrome) was significantly increased in the diabetic TGFβ1 TG mice compared to the diabetic WT mice and non-diabetic TGFβ1 TG mice. The area stained with collagen was also significantly enhanced in the diabetic WT mice compared to the non-diabetic WT mice (Figure 7C,D).

### 2.8. Elevated Renal Expression of Fibrosis Markers in Diabetic TGFβ1 TG Mice

**Figure 8:**
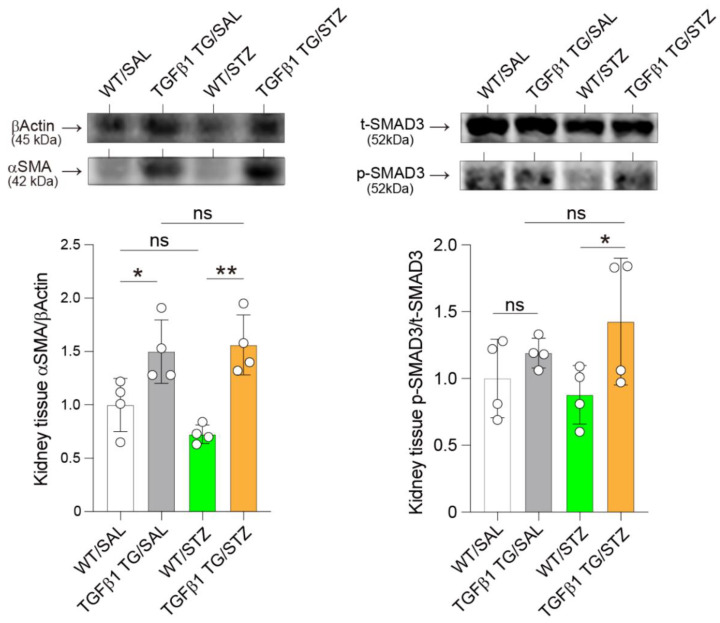
Increased expression of αsmooth muscle actin and phosphorylation of SMAD3 in diabetic transforming growth factorβ1 mice. The protein expression of αsmooth muscle actin (αSMA) and the activation of SMAD3 were assessed by western blotting. *n* = 4 in each group. Data are the mean ± S.D. Statistical analysis by ANOVA with Newman–Keuls’ test. * *p* < 0.05; ** *p* < 0.01.

The protein expression of α-SMA and activation of SMAD3 were assessed by western blotting. The protein expression of αSMA was significantly increased in the diabetic TGFβ1 TG mice compared to the diabetic WT mice. The non-diabetic TGFβ1 TG mice also showed a significantly increased level of αSMA in the renal tissues compared to their non-diabetic WT counterparts. The phosphorylation of SMAD3 was significantly increased in the diabetic TGFβ1 TG mice compared to the diabetic WT mice. No significant difference was observed between the diabetic and non-diabetic WT mice (Figure 8).

## 3. Discussion

In the present study, we found that the overexpression of TGFβ1 in the kidneys is associated with insulin resistance and the rapid progression of glomerulosclerosis under diabetic conditions in mice.

A worse clinical course and poor prognosis have been reported in patients with diabetic nephropathy compared to non-diabetic nephropathy [8,9,10]. The abnormal circulating glucose levels and their metabolic consequences may contribute to this poor clinical outcome in patients with DM-associated chronic kidney disease [7]. However, the mediating factor is unclear. Previous human studies have shown systemic levels of TGFβ1 increase in type-1 and type-2 DM, obesity with insulin resistance, and diabetic nephropathy [11,21,34,35,36,37,38,39]. Evidence from experimental animal and in vitro studies also recapitulated the pathogenic role of TGFβ1 in diabetic nephropathy [40,41,42,43]. An important factor that triggers the enhanced expression of TGFβ1 in the kidneys is hyperglycemia [44,45,46,47,48]. Even a transient increase in the blood glucose levels has been shown to enhance the urinary levels of TGFβ1 in humans [49]. Hyperglycemia can stimulate TGFβ1 expression by inducing the activation of the human TGFβ1 promoter through the AP-1 transcription factor under the regulation of the protein kinase C and p38 mitogen-activated protein kinase pathways or by transcriptional activation induced by a putative glucose-responsive element present in the TGFβ1 promoter [45,47]. In addition to enhancing the production of TGFβ1, hyperglycemia can also promote the inflammatory response by activating the macrophages through the TGFβ1-activated kinase (TAK1) that induces the release of pro-inflammatory cytokines by NFκB activation [50]. In addition, insulin resistance combined with hyperinsulinemia and hyperglycemia has been reported to upregulate the expression of TGFβ1 in the kidneys in the pre-diabetes phase in humans [36,49,51]. All types of resident renal cells and infiltrating inflammatory cells can act as sources and exacerbate the release of TGFβ1 in the pre-diabetes phase [19,36,49,51]. In the present study, we induced a DM state in WT mice and TGFβ1 TG mice overexpressing the human TGFβ1 in the kidneys and evaluated whether hyperglycemia affected the circulating levels of TGFβ1. We found that the blood levels of active TGFβ1 were significantly increased in the diabetic TGFβ1 TG (TGFβ1 TG/STZ) compared to the non-diabetic TGFβ1 TG (TGFβ1 TG/SAL) mice and diabetic WT mice (WT/STZ), suggesting an enhanced TGFβ1 activation in the presence of hyperglycemia. Cells synthesize and secrete TGFβ1 as a latent protein [52]. The activation of the latent form of TGFβ1 may be facilitated by commonly hyperglycemia-associated conditions, including metabolic acidosis, oxidative stress, and the increased expression of thrombospondin-1, integrins, or plasmin [53,54,55,56,57,58].

Another important finding of the present study is the accelerated progression of renal dysfunction in the diabetic TGFβ1 TG (TGFβ1 TG/STZ) mice compared to the non-diabetic TGFβ1 TG (TGFβ1 TG/SAL) mice and diabetic WT (WT/STZ) mice. The plasma levels of the renal dysfunction markers BUN and creatinine were not significantly different between the diabetic and non-diabetic TGFβ1 TG mice at weeks 2 or 6, but they were significantly increased in the diabetic TGFβ1 TG mice at week 9 compared to the non-diabetic TGFβ1 TG mice. In addition, while the increase in the plasma levels of BUN and creatinine was remarkably high in the diabetic TGFβ1 TG mice at week 9 compared to week 6, the increase was weak in the non-diabetic TGFβ1 TG mice and diabetic WT mice. The only difference between the TGFβ1/SAL and TGFβ1 TG/STZ groups was the presence of hyperglycemia with high circulating levels of active TGFβ1. Therefore, we speculate that the explanation for the significant and rapid progression of renal dysfunction in the TGFβ1 TG/STZ group compared to the TGFβ1 TG/SAL group and diabetic WT mice is the presence of hyperglycemia with a high circulating level of active TGFβ1. In line with this finding, the collagen deposition area in the kidneys was significantly increased in the diabetic TGFβ1 TG mice compared to the non-diabetic TGFβ1 TG mice and diabetic WT mice. A potential explanation for this is the increased accumulation of collagen-producing αSMA-positive myofibroblasts induced by the activation of the TGFβ1/SMAD3 signal pathway [11]. The increased SMAD3 phosphorylation and αSMA expression in the kidneys, compared to the WT/STZ mice, supports this explanation. Overall, these results support, at least in part, the role of TGFβ1 as a mediator of accelerated and progressive nephropathy under diabetic conditions. However, it is worth noting that the expression of αSMA and SMAD3 phosphorylation in the kidneys was not affected by the difference in the circulating levels of active TGFβ1 between the diabetic and non-diabetic TGFβ1 TG mice. A probable explanation for this observation is the short-term follow-up. In our present study, all the mouse groups were followed-up for only 9 weeks. Therefore, an experimental protocol with a longer follow-up period would probably be required to detect significant changes in αSMA and phosphorylated SMAD3 in the kidneys between diabetic and non-diabetic TGFβ1 TG mice. Future studies should address this important question.

TGFβ1 may also worsen the diabetic state by decreasing the sensitivity to insulin. Several cases of evidence support this assumption. For example, TGFβ1 and its intracellular SMAD signaling pathway have been associated with insulin resistance in women with polycystic ovary syndrome [59,60,61]. Other researchers observed that SMAD knockout mice were protected from diet-induced obesity and DM, that elevated concentrations of TGFβ1 increases the risk of developing type-2 DM, that SMAD proteins are involved in obesity-induced glucose, and that lipid abnormalities support the role of TGFβ1 signaling in the pathogenesis of insulin resistance and DM [22,26,62,63,64]. In particular, the activation of SMAD3 appears to play a critical role in TGFβ1-mediated insulin resistance [65,66]. In addition, TGFβ1 may also contribute to insulin resistance through its modulatory activity on SMAD-independent pathways, including mitogen-activated protein kinases and Akt [67,68,69]. The remodeling of the extracellular matrix by TGFβ1 in the skeletal muscles may also contribute to insulin resistance. A physical barrier created by enhanced collagen deposition in the endomysium, epimysium, and basement membrane in the muscles may impair glucose uptake and the binding of insulin to its cell surface receptor, leading to insulin resistance [65,70,71,72,73,74]. The persistently high blood glucose levels, the abnormal glucose tolerance test, and the increased HOMA-IR values in the diabetic TGFβ1 TG mice compared to the non-diabetic TGFβ1 TG mice and diabetic WT mice and the inhibitory effect of TGFβ1 on insulin activity in the hepatocyte and myotubes observed in the present study reinforce the role of TGFβ1 in insulin resistance.

Another important observation that supports the role of TGFβ1 overexpression in insulin resistance in the present study is the significant difference in the glucose levels between the diabetic TGFβ1 TG mice and diabetic WT mice. In the present study, we induced diabetes following a universally accepted experimental protocol (one intraperitoneal injection per day of STZ for five consecutive days) [75,76,77]. The destructive effect of STZ on the pancreatic islets was moderate in both the WT/STZ and TGFβ1 TG/STZ groups, and there was no significant difference in the plasma insulin levels between the WT/STZ and TGFβ1 TG/STZ groups. However, despite this moderate destructive effect of STZ, mice from the WT/STZ group became significantly diabetic, as shown in Figure 2A (significantly increased glucose levels in WT/STZ versus WT/SAL at weeks 3 and 5 after STZ injection) and Figure 2B (significantly increased glucose levels in WT/STZ versus WT/SAL after 15 and 120 min during the glucose tolerance test). In addition, despite the lack of significant differences in the pancreatic islet area and plasma insulin levels between the WT/STZ and TGFβ1 TG/STZ mice, there was a dramatic and significant increase in the weekly glucose levels (weeks 3, 5, and 7 in Figure 2A) and in the glucose levels during the glucose tolerance test (30 and 60 min in Figure 2B) in the TGFβ1 TG/STZ group compared to the WT/STZ group. What is the explanation for the worse diabetic condition of the TGFβ1 TG/STZ mice compared to the WT/STZ mice? The only reasonable explanation for the significant difference in the glucose levels after the STZ injection and during the glucose tolerance test between the TGFβ1 TG/STZ and WT/STZ groups is the increased circulating TGFβ1-associated insulin resistance, as confirmed by the significantly increased HOMA-IR in the TGFβ1 TG/STZ mice compared to the WT/STZ mice, as described in Figure 2C. These findings further support the detrimental effect of TGFβ1 overexpression on insulin sensitivity under diabetic conditions.

**Figure 9:**
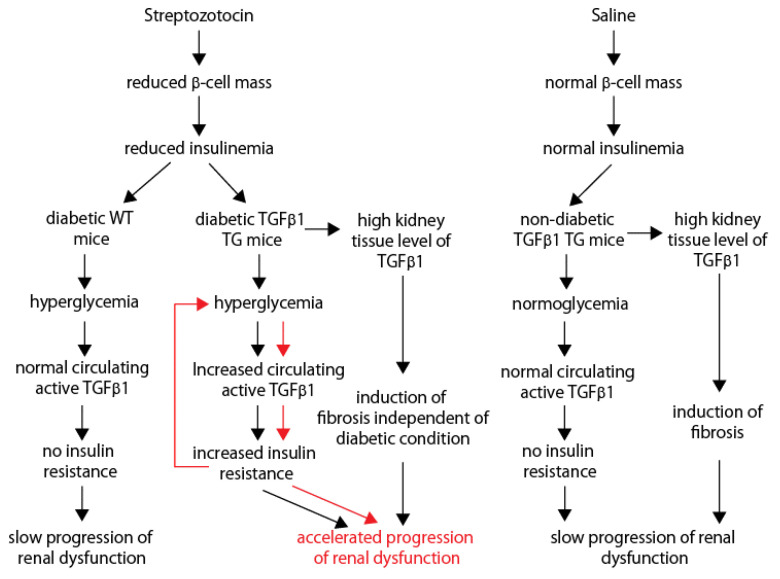
The vicious cycle of hyperglycemia, in which increased circulating active TGFβ1 and insulin resistance may explain the accelerated progression of renal dysfunction in diabetic TGFβ1 TG mice. Red arrows indicate the vicious cycle.

A critical question that needs to be addressed is: to what extent can renal dysfunction be attributed to the direct effect of hyperglycemia on the diabetic TGFβ1 TG mice due to insulin resistance (and the related glucotoxicity) compared to the increased circulating active TGFβ1 levels? Based on the results of our present study, we speculate that the following sequential events may explain the rapidly progressive renal dysfunction in diabetic TGFβ1 TG mice: a reduction in the islet β-cell mass by STZ induces a vicious cycle, where hyperglycemia increases the circulating levels of active TGFβ1, which further worsens hyperglycemia by inducing insulin resistance (Figure 9). Consequently, the high level of TGFβ1 in the kidneys and the high circulating level of active TGFβ1 lead to accelerated renal dysfunction in diabetic TGFβ1 TG mice. Non-diabetic TGFβ1 TG mice and diabetic WT mice show no high levels of circulating active TGFβ1 and, thus, the course of renal dysfunction is slow.

## 4. Materials and Methods

### 4.1. Animals

A TGFβ1 transgenic (TG) mouse in a C57BL/6J background overexpresses, specifically in the glomeruli (podocytes), the full-length human TGFβ1 gene under the control of the mouse podocin promoter [18]. To generate the TG mouse, a chimeric podocin TGFβ1 bacterial artificial chromosome transgenic construct that harbors the full-length coding exons and an intervening intron of human TGFβ1 in place of the mouse podocin gene locus by bacterial artificial-chromosome-recombination-mediated genetic engineering was prepared. The transgenic mouse was then generated by the pronuclear injection of the chimeric construct into C57BL/6J mouse embryos. The transgenic founders were confirmed by southern blotting [18]. The TGFβ1 TG mice developed spontaneously progressive and fatal kidney fibrosis. The C57BL/6 mice were provided by Nihon SLC (Hamamatsu, Japan) [18]. Male 8- to 10-week-old WT and TGFβ1 mice were used in all the experiments. The breeding was performed in the animal laboratory of Mie University in a pathogen-free environment at 25 °C, with a humidity of about 50%. The mice were subjected to a light/dark cycle of 12 h each and provided with food and water ad libitum. The experimental protocols were approved by the Committee on Animal Investigation of Mie University (approval no. 27-4; date: 19 August 2015), and all the procedures were carried out according to the institutional guidelines.

### 4.2. Induction of Experimental Diabetes

The WT and TGFβ1 TG mice were intraperitoneally injected with STZ (Sigma, St. Louis, MO, USA) to develop diabetes. STZ was administered at a dose of 40 mg/kg body weight for five consecutive days [76]. In addition, a group of mice that received an intraperitoneal injection of saline (SAL) were used as the controls. The mice were allocated to four experimental groups: WT mice that received intraperitoneal STZ (WT/STZ) or SAL (WT/SAL), and TGFβ1 TG mice that received intraperitoneal STZ (TGFβ1 TG/STZ) or SAL (TGFβ1 TG/SAL). All the mice that received an injection of STZ or SAL were included in the evaluation of hyperglycemia.

### 4.3. Evaluation of Glucose Parameters

The blood levels of glucose were measured weekly after the intraperitoneal administration of STZ. The glucose tolerance test was performed in the 4th week after STZ injection as follows: mice that fasted overnight received an intraperitoneal injection of glucose at a dose of 1 g/kg, and the blood glucose levels were measured after 0, 15, 30, 60, and 120 min, as previously described [78]. The glucose-stimulated insulin secretion test was performed after 16 h of fasting by an intraperitoneal injection of glucose at a dose of 3 g/kg of the mouse body weight. Blood was collected from the tail vein at 0, 2, 10, and 30 min after the glucose injection. The blood glucose level was measured by the glucose oxidase method, and the blood insulin concentration was measured using an ALPCO measuring instrument kit (Salem, NH, USA).

### 4.4. Histological Study

An overdose of isoflurane was used to euthanize the animals, followed by exsanguination nine weeks after the STZ injection. The pancreas and kidney tissues were incised, dehydrated, embedded in paraffin, and cut into 3 µm-thick sections for hematoxylin and eosin, periodic acid–Schiff, or Masson’s trichrome staining following standard methods. The slides were observed under an optical microscope BX53, and several microphotographs were taken using a DP73 digital camera with DP controller software (Olympus, Tokyo, Japan). The area of pancreatic islets stained with hematoxylin and eosin was measured, and the total area was calculated in relation to the control group [32,79]. Glomerular sclerosis was evaluated as previously described [18]. Briefly, 10 glomeruli per mouse were randomly taken and scored according to the PAS-positive area based on the following criteria: score 0: normal glomeruli, score 1: mild mesangial thickness (PAS positive area < 25%), score 2: moderate segmental sclerosis (PAS positive area 25–50%), score 3: severe segmental sclerosis (PAS positive area 50–75%), and score 4: global sclerosis (PAS positive area ≥ 75%). Each mouse’s average score of 10 glomeruli was considered as the glomerular sclerosis score. The scoring was performed by 8 investigators blinded to the treatment groups. The grade of kidney fibrosis was estimated using tissue samples stained with Masson’s trichrome. Five microphotographs of the glomeruli per mouse were randomly taken, and the percentage of fibrosis was evaluated. The Masson-trichrome-positive area/glomerulus area ratio was calculated using WinROOF image processing software (Mitani Corp., Fukui, Japan), and the fibrosis grade was compared between groups, as previously described [18]. An investigator blinded to the treatment group performed the test using a BX50 microscope with a plan objective, combined with an Olympus DP70 digital camera (Tokyo, Japan) and the WinROOF image processing software (Mitani Corp., Fukui, Japan).

### 4.5. Cell Culture

L6 rat skeletal myoblasts, provided by Hitoshi Ashida, Kobe University, and HepG2 cells (RIKEN Cell Bank, Ibaraki, Japan) were cultured in Dulbecco’s modified Eagle’s medium (DMEM; Sigma-Aldrich) containing 10% (*v*/*v*) heat-inactivated fetal calf serum (FCS) in a humidified atmosphere at 37 °C with 5% CO_2_. The L6 rat skeletal myoblasts were fused into myotubes by changing the media to DMEM supplemented with 2% FBS for 2–4 days after confluence. The L6 and HepG2 cells were incubated in DMEM + 1% BSA for 6 h and placed in DMEM + 1 g/L glucose immediately before the addition of TGFβ1. Then, 30 min after the TGFβ1 treatment, insulin (200 nM) was added, and the cells were cultured for 4 h. The Glucose Colorimetric Assay Kit (BioVision) was used to measure the glucose content of the culture supernatant.

### 4.6. Biochemical Analysis

The plasma creatinine levels were measured by an enzymatic method and blood urea nitrogen was measured by a colorimetric method (NCalTM NIST-Calibrated kit; Arbor Assays, Ann Arbor, MI, USA) according to the manufacturer’s instructions. The plasma concentrations of total and active TGFβ1 were measured using a commercially available immune assay kit (R&D Systems, Minneapolis, MN, USA) following the manufacturer’s instructions. The homeostasis model assessment for insulin resistance (HOMA-IR) was determined as follows: HOMA-IR = [fasting insulin (µU/mL) × fasting glucose (mmol/L)]/22.5 (17).

### 4.7. Western Blotting

Standard methods were used to perform western blotting using antibodies against phosphorylated SMAD3, total SMAD3, α-smooth muscle actin (α-SMA), and β-actin from Cell Signaling (Danvers, MA, USA).

### 4.8. Statistical Analysis

Data are expressed as the mean ± standard deviation (S.D.) unless otherwise specified. The distribution of all the parameters was evaluated using the Shapiro–Wilk test. Statistical differences between variables with a normal distribution were evaluated by one-way analysis of variance (ANOVA) with the Newman–Keuls’ test. Variables with a skewed distribution were evaluated by Kruskal–Wallis one-way ANOVA with Dunn’s test. For the statistical analysis, we used Graph-pad Prism version 9.0 (San Diego, CA, USA). A *p* < 0.05 was considered significant.

## 5. Conclusions

This study shows that increased circulating levels of TGFβ1 under diabetic conditions are associated with the increased activation of TGFβ1, insulin resistance, and accelerated progression of kidney fibrosis and renal dysfunction.

## Data Availability

All the data obtained during the current study are available from the corresponding author upon reasonable request.
